# Preparation and Drug Release Properties of a Thermo Sensitive GA Hydrogel

**DOI:** 10.3390/polym13010119

**Published:** 2020-12-30

**Authors:** Jiufang Duan, Yirong Huang, Shiyu Zong, Jianxin Jiang

**Affiliations:** MOE Engineering Research Center of Forestry Biomass Materials and Bioenergy, Beijing Forestry University, Beijing 100083, China; huangyirong@163.com (Y.H.); zongshiyu@126.com (S.Z.); jiangjx@bjfu.edu.cn (J.J.)

**Keywords:** galactomannan, drug carrier, hydrogel, pH-responsive, thermo-responsive

## Abstract

A high-strength galactomannan (GA)-based hydrogel with thermal response and pH response is introduced in this paper. GA, N-isopropylacrylamide (NIPAM), N-[3-dimethylamino)propyl]methylacrylamide (DMAPMA), and montmorillonite were used to form hydrogels through a simple mixed static system. Fourier-transform infrared spectroscopy (FTIR), differential scanning calorimetry (DSC), and scanning electron microscopy (SEM) were used to characterize the structure and properties of the hydrogels. The compressive strength of the the hydrogel increased from 23.9 to 105.61 kPa with the increase of GA dosage from 0 to 1.5 wt%. When the NIPAM content in the monomer increased from 75% to 95%, the lower critical solution temperature (LCST) of the hydrogel changed from 36.5 to 45.8 °C. When the monomer content was higher than 10wt%, the swelling kinetics of the sample changed from the second-order equation to the first-order equation. With the increase of the proportion of NIPAM monomer, the release rate of bovine serum album in the early stage was faster, and the cumulative release rate was close to 100%.The release rate of bovine serum albumin at 37 °C was higher than that at 25 °C. The release rate of the hydrogel containing bovine serum albumin was the fastest under the condition of pH 7.4, followed by those at pH 6.6 and pH 5.0. The results showed that this thermal-responsive hydrogel has potential applications as a drug carrier for colon delivery.

## 1. Introduction

Galactomannan (GA) is a kind of green, environmental protection and cheap raw material. It is a kind of water-soluble polysaccharide composed of galactose and mannose residues. GA is composed of a 1,4-β-D-mannose main chain, and a single D-galactose unit is connected to the main chain by an α-(1,6)-glycosidic bond. Due to different plant sources of polysaccharides, the ratio of mannose to galactose varies from 1:1 to 4:1 [1]. The modification of GA mainly includes surface modification, grafting, formation of double network structure, and enzyme method and metal crosslinking method modification [2,3,4,5]. The GA-based hydrogel is an important part of hydrogel research. It is mainly used in water treatment and energy storage [6], wound dressing [7,8], biomedical [5,9], flexible sensor [10], adsorbent [11], drug release [12], agricultural water and soil conservation [1], and other fields [13,14,15].

Stimuli-responsive hydrogels can change rapidly when suffering environmental stimuli, such as the temperature, pH, light, mechanical force, and magnetic field, which respond to the thickness, expansion, color, size, and/or other physical properties of materials. Stimulus-responsive polymers have attracted a great deal of attention due to their stimulus-response behavior and potential applications in drug delivery, tissue engineering, biosensors, smart coatings, biomimetic devices, and so on. In these materials, shape-sensitive and temperature-sensitive multifunctional intelligent-response hydrogels haveattracted more and more attention because of its potential biomedical applications. For example, shape memory properties of hydrogels are erasable and depend on external conditions such as pH, light, and heat [16,17,18,19]. It has been used in various high-tech fields, such as sensors, artificial muscles, wearable devices, actuators, soft robot technology, and biomedicine. Temperature-sensitive materials have also been extensively studied, such as natural polysaccharide guar gum self-assembled peptide hydrogels, which have characteristics of elastic, thermo-responsive, self-healable, and so on. It can be used in 3D printing, wound healing, suture, and other fields [20]. The Pd-NPs-loaded guar hydrogel with sol-gel transition, which is achieved by a simple heating/cooling cycle, is expected to be used in a new catalytic system [21]. The lower critical solution temperature (LCST) of poly(N-isopropylacrylamide) (PNIPAM) in pure water is 32 °C, which is close to the body temperature [22]. Therefore, PNIPAM has broad application prospects in biomedical materials and is the most widely studied temperature-sensitive material. In [23], GA–g–PNIPAM copolymer nanoparticles was prepared, and the influence of the molar weight of GA on the particle size of the grafted PNIPAM copolymer was investigated. It was found that this material has the potential as a nanocarrier.

The mechanical strength of the PNIPAM hydrogel is relatively low, which limits its application [22,23]. GA has a high molecular weight, good water solubility, and a hydroxyl-rich side chain, which may increase the degree of intramolecular crosslinking and overall network entanglement, thus affecting the mechanical properties of materials [24]. GA-based hydrogels with adjustable mechanical properties are expected to provide new materials for the field of regenerative medicine and tissue engineering. GA-based hydrogel delivery carriers can be degraded by colon microorganism and have controllable and fixed-point drug delivery properties [25,26].

In this study, we have developed a GA-based hydrogel with high mechanical properties, which can be adjusted in thermo-responsive properties by virtue of the dual advantages of GA polyhydroxyl structure and thermo-sensitive properties of PNIPAM. The mechanical properties, thermal response properties, pH response properties, and drug release behavior of hydrogels were systematically investigated.

## 2. Material and Methods

### 2.1. Material

N-isopropylacrylamide (NIPAM), DMAPMA, GA, N,N-methylene bisacrylamide(MBA), montmorillonite, BSA, ammonium persulfate (APS), sodium chloride, sodium tetraborate, and tetramethylethylenediamine were obtained from Beijing Lanyi Chemical Products Co., Ltd. (Beijing, China) and used as received.

### 2.2. Preparation of the GA–P(NIPAM-co-DMAPMA) Hydrogel

First, 0.05 g GA was dissolved in 10 mL deionized water and stirred evenly to prepare a guar gum solution, and 1.075 g NIPAM, 0.086 g DMAPMA, 0.012 g MBA, and 0.2 g montmorillonite were stirred evenly. Then, APS and tetramethylethylenediamine was added under the ice bath condition and stirred evenly. The solution was poured into the corresponding mold to form a hydrogel by placing it at room temperature for 12 h. The specific experimental scheme is shown in the Table 1.

### 2.3. Methods

#### 2.3.1. Morphology Characterization

A small amount of the GA–P(NIPAM-co-DMAPMA) hydrogel sample was freeze-dried and adhered to a conductive adhesive. After spraying gold, the samples were placed in a scanning electron microscope (SEM) sample bin and observed with an SEM (S-3400N, Hitachi (HIACHI), Tokyo, Japan).

#### 2.3.2. FTIR

The sampleswere placed in an oven and dried at 120 °C. The samples were mixed with potassium bromide to grind and compressed. A Fourier-transform infrared spectrometer (tensor 27, Bruker, Ettlingen, Germany) was used to determine the infrared spectrum. The scanning range was 400–4000cm^−1^. The Scanning number was 32 times, and the resolution was 2 cm^−1^.

#### 2.3.3. Differential Scanning Calorimetry (DSC) Characterization

The lyophilized samples were tested by a differential thermal scanner (20F3, NETZSCH, Selb, Germany) in N_2_ atmosphere.

#### 2.3.4. Characterization of UV Spectrum

The samples were placed in a10 mL glass colorimeter. The absorbances of the polymers at different temperatures were determined by an ultraviolet visible spectrophotometer (UV-2000, Shanghai United Instrument Co., Ltd., Shanghai, China) at a 500 nm wavelength.

#### 2.3.5. Characterization of Mechanical Properties

A cylindrical gel sample (φ: 20 mm; L: 25 mm) with a uniform size was prepared by hydrogels and tested by a universal testing machine (3365, Inter dragon, Norwood, MA, USA). The crosshead speed was 2 mm/min.

#### 2.3.6. Swelling Test of the Hydrogels

The sample was placed in an oven at 120 °C and dried until absolutely dry. Subsequently, the sample weight m_1_ was weighed and recorded. Then, the sample was immersed in deionized water at different temperatures. The gel was removed from water regularly, and the surface moisture was dried with a filter paper and then weighed. The sample mass m_2_ was recorded at time t. The swelling ratio can be calculated according to the following equation:

Swelling ratio (%) = (m_2_ − m_1_)/m_1_ × 100%. (1)

#### 2.3.7. Loading and Release of Bovine Serum Protein

In a range of 0–1.0 mg/mL BSA, PBS buffer solutions with pH values of 5, 6.6, and 7.4 were used as solvents. The wavelength of the UV/VIS spectrophotometer was 280 nm, and the size of a quartz cuvette was 10 mm. The relationship between BSA concentration and absorbance was obtained.

BSA with a mass of 0.2 g was weighed and dissolved in 100 mL deionized water. Ten milliliters of the BSA solution was transferred into a centrifuge tube with a pipette gun.

Thirty mg of GA–P(NIPAM-co-DMAPMA) composite hydrogel, which was frozen and dried, was immersed in 10 mL of a 2 mg/mL BSA aqueous solution. Then, it was placed in a constant temperature oscillator at 25 and 37 °C, and the rotation speed was 130 rpm. After shaking for 24 h, a centrifuge was used at 4000 r/min for 10 min. The sediment was weighed after freeze-drying. The volume of a supernatant was fixed to a certain volume, and the absorbance was measured at 280 nm. After comparing the standard curve in water with BSA, the amount of BSA in the supernatant solution was converted and the drug loading rate and entrapment efficiency of BSA were calculated.

The release experiment was carried out in a constant temperature oscillator (oscillating rate of 130 r/min) at different temperatures (25 and 37 °C): 30 mg drug-loaded gels were dispersed in different buffer solutions of 80mL (pH = 5.0, 6.6, and 7.4) at 0.5, 1.5, 2.5, 5.5, 7.5, 9.5, 13.5, 24, 29, 35, and 48 h. Five mL of the sustained-release solution each time was taken, and 5 mL of fresh buffer solution was added. Subsequently, the sustained release liquid was measured at a 280 nm wavelength, and the absorbance A was measured by a 10 mm quartz colorimeter. It was substituted into the standard curve, and the release rate of BSA was calculated. The calculation equation was written as follows:

(2) $$\mathrm{Cumulative}\;\mathrm{release}\;\mathrm{amount}\;(\%)=\frac{V_{e}\sum_{1}^{n-1}C_{n-1}+V_{0}C_{n}}{m_{\mathrm{drug}}}\times 100\%,$$

where V_e_ is the displacement volume of BSA, C_n_ is the concentration of the release solution during the nth displacement sampling, V_0_ is the total volume of the released medium, and m_drug_ is the total mass of the drug contained in the sample hydrogel.

## 3. Results and Discussion

### 3.1. Synthesis and Characterization of the GA–P(NIPAM-co-DMAPMA) Hydrogel

**Figure 1:**
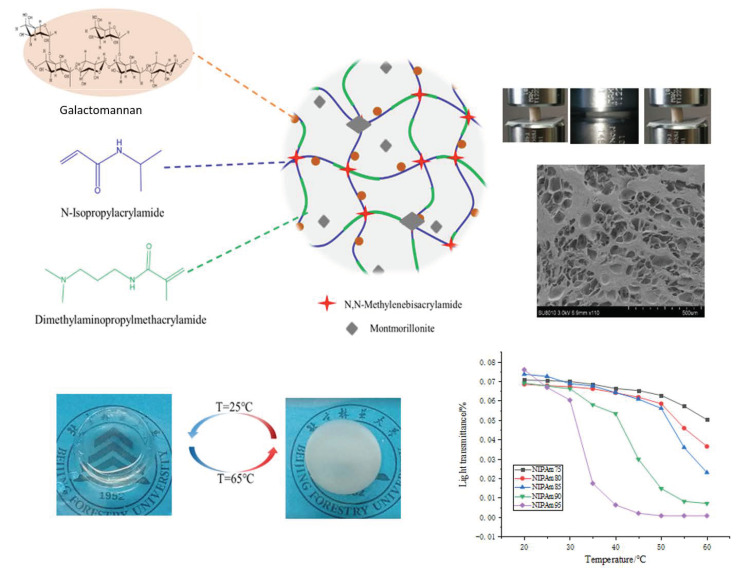
Scheme of the GA–P(NIPAM-co-DMAPMA) hydrogel structure and properties.

In this study, a GA–P(NIPAM-co-DMAPMA) hydrogel was prepared by radical graft polymerization of GA, NIPAM, and DMAPMA in an aqueous solution. Montmorillonite with a layered structure was used as a physical crosslinking agent. DMAPMA was selected as a comonomer to adjust the LCST of copolymers to obtain materials with different service temperatures. The mechanism of GA participating in chemical reaction is shown in Figure S1 [3]. Carbonyl oxygen in hydrogel molecules can form hydrogen bonds with hydroxyl groups and amino groups. Hydrogen bonds and covalent chemical bonds formed a three-dimensional network of the GA–P(NIPAM-co-DMAPMA) hydrogel. The GA–P(NIPAM-co-DMAPMA) hydrogel showed porous honeycomb morphology (Figure 1). The GA–P(NIPAM-co-DMAPMA) hydrogel had a temperature response.

Figure 1 shows the change of transmittance of the series hydrogel with temperature. The light transmittance of the samples was highest at 20 °C, which gradually decreased with the increase of temperature, and decreased sharply near the corresponding LCST. The higher the proportion of NIPAM in the monomer, the more obvious the change of transmittance and the higher temperature sensitivity is. At lower temperatures, the hydrogel was a transparent elastic solid. When the temperature was higher, the hydrogen bond in the hydrogel, which was formed by amide groups and water molecules, was broken. The isopropyl group exerted its hydrophobic effect to make the segment close to the chain and reunite, so the transparency decreased and the opaque state occurred [27]. The LCST was a critical value. At 25 °C (T < LCST), it showed a transparent state. It turned white at 65 °C (T > LCST), the size of the sample shrank, and the transparency of the hydrogel was reversible. The GA–P(NIPAM-co-DMAPMA) hydrogel had both a hydrophilic group (amide group) and a hydrophobic group (isopropyl group). Under this temperature, the hydrophilic amide group produced a hydrophilic force with water and formed hydrogen bonds, so that the PNIPAM chain was hydrophilic and stretched in the aqueous solution. At this time, the hydrogel was colorless and transparent. When the temperature gradually increased, the entropy drove the hydrophobic interaction in water and the hydrophobic residue dehydrated at high temperature [28,29,30]. The hydrophobic isopropyl group gradually played a role, which made the hydrophobic force increase continuously. When the temperature was higher than the LCST, the polymer chains absorbed energy and gathered together; the hydrogen bond with water was broken completely, and the polymer chains were white and opaque.

**Figure 2:**
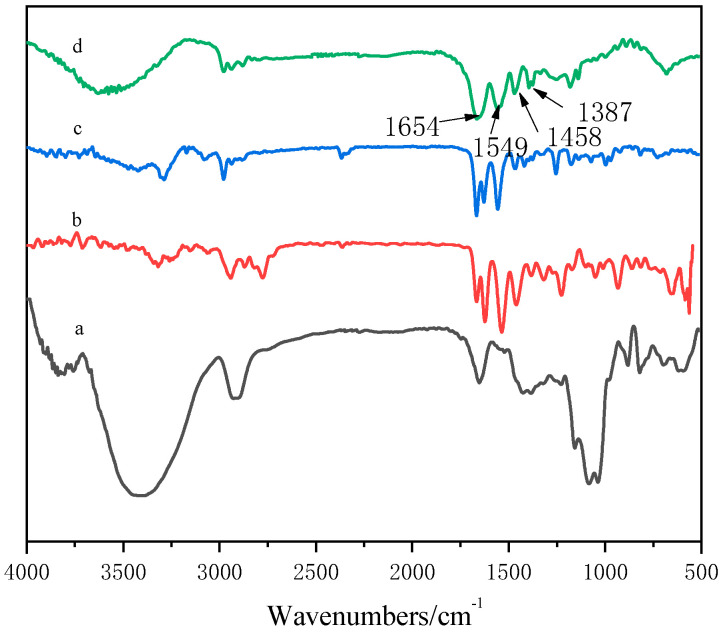
FTIR spectra of GA–P(NIPAM-co-DMAPMA) hydrogels. (**a**) GA; (**b**) DMAPMA; (**c**) NIPAM; (**d**) GA–P(NIPAM-co-DMAPMA) hydrogel.

It can be seen from Figure 2a that the absorption peak near 3480 cm^−1^ was the OH stretching vibration on GA, the absorption peak near 2924 cm^−1^ was the saturated CH stretching vibration peak of –CH_2_ in the sugar unit, the ring vibration absorption peak of galactose or mannose was about 1653 cm^−1^, and the stretching vibration absorption peak of C–O–C appeared at 1022 cm^−1^ [31].Figure 2b shows that the absorption peak at 547.7 cm^−1^ referred to the N–H deformation vibration on the secondary amide, the absorption peak at 3074 cm^−1^ was the multiple frequency of the N–H deformation vibration, the absorption peak at 3281 cm^−1^ was the N–H stretching vibration on the amide group, and the absorption peak at 2975 cm^−1^ was the stretching absorption peak of C–H. Figure 2c shows that the absorption peak at 3320 cm^−1^ was the N–H stretching vibration absorption peak of amide, the absorption peak at 2940 cm^−1^ was the asymmetric stretching vibration absorption peak of CH_3_, the absorption peak at 2865 cm^−1^ was attributed to the symmetric stretching vibration of CH_3_, the absorption peak at 1656 cm^−1^ belonged to the stretching vibration of C=O of amide, the absorption peak at 1536 cm^−1^ belonged to the stretching vibration of C–N, and the absorption peak at 1311 cm^−1^ was the characteristic absorption peak of the tertiary amine group. In the copolymer spectrum (Figure 2d), a wide stretching vibration absorption band of –OH appeared at 3602 cm^−1^. Compared with the results shown in Figure 2a, the –OH peak shifted to a higher wave number, which may be due to the formation of hydrogen bonds in the copolymer.

In the spectrum of the GA–P(NIPAM-co-DMAPMA) hydrogel, the following absorption peaks appeared: bending vibration peak of secondary amide CO at 1654 cm^−1^, variable angle vibration peak of secondary amide C–N–H at 1549 cm^−1^, C–H asymmetric bending vibration peak of CH_3_ and CH_2_ at 1458 cm^−1^.The symmetry bending vibration peaks of isopropyl were at 1387 and 1367 cm^−1^. The results indicated that NIPAM and DMAPMA were successfully introduced into the GA hydrogen system through a free radial reaction in an aqueous solution.

### 3.2. Mechanical Strength of the Hydrogel

**Figure 3:**
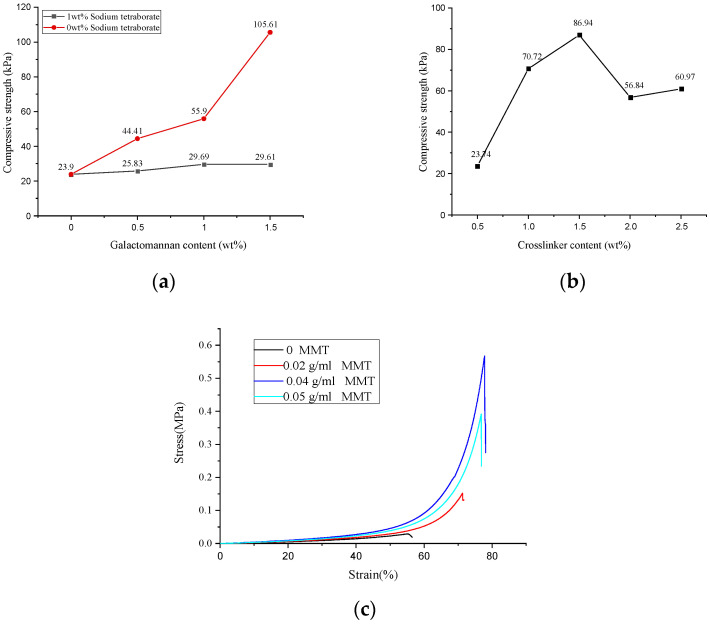
Mechanical strengths of the GA–P(NIPAM-co-DMAPMA) hydrogels with (**a**) different GA contents and (**b**) crosslinking agent contents. (**c**) Hydrogel compression stress–strain curves for different montmorillonite contents.

The mechanical properties of the hydrogel determine its application and service life. As shown in Figure 3a, when the dosage of GA increased in a range of 0–1.5 wt%, the compressive strength of the GA–P(NIPAM-co-DMAPMA) hydrogel changed a little. However, when we immersed the GA–P(NIPAM-co-DMAPMA) hydrogel into a 0.026 mol/L four sodium borate solution for 0.5 h, it was found that the compressive strength of the hydrogel increased from 23.9 to 105.61 kPa with the increase of GA dosage from 0 to 1.5 wt%. This is due to the cis hydroxyl group on the carbohydrate residues of the GA main chain and crosslinking with boric acid to form a network structure to enhance the strength of the hydrogel [1].

The compressive strengths of the hydrogel first increased and decreased with increasing crosslinking agent concentration. When the crosslinking agent content was 1.5 wt%, the maximum compressive strength of the hydrogel was 86.94 kPa. When the crosslinking agent content was less than 1.5 wt%, the crosslinking density increased, so that the compressive strength of the hydrogel increased (Figure 3b). A significantly large amount of the crosslinking agent led to a higher crosslinking density, which made it difficult to produce a relative displacement between the polymer chains of the hydrogel. Therefore, the hydrogel exhibited more brittle, lower toughness, and lower compressive strength [23].

When the montmorillonite contents were 0, 0.02, 0.03, 0.04, and 0.05 g/mL, the maximum compressive strengths of the hydrogel were 28.60, 152.16, 301.86, 567.93, and 392.18 kPa (Figure 3c). Montmorillonite nanosheets in a solution provided more interlacing and winding structures for polymer molecular chains. The physical crosslinking of the montmorillonite was conducive to the formation of the complex network structure of the polysaccharide hydrogel. Therefore, it showed good compression performance on the macrolevel.

After investigating the relationship between the content of each component and the compressive strength, the optimal content of the GA–P(NIPAM-co-DMAPMA) hydrogel was obtained. The content of GA was 1.0 wt%, the amount of the crosslinking agent was 1.5 wt%, the dosage of APS was 2 wt%, the content of vinyl monomer (NIPAM and DMAPMA) was 12 wt%, and the content of montmorillonite was 0.04 g/mL (Figure 3 and Figure S2). The possible reason is that when the content of GA was higher than 1.0 wt%, the viscosity of the solution increased sharply and it is difficult to mix the reaction components evenly. When the crosslinking agent dosage was higher than 1.5 wt%, the crosslinking density was significantly high, resulting in the decrease of hydrogel strength. When the content of the initiator was higher than 2 wt%, a large number of free radicals were generated. The molecular weight of the polymer was low, and the polymerization chain was short, resulting in an uneven network structure and a reduced compressive strength of the hydrogel. When the monomer content was very high, a large number of free radicals were produced in a short time. Therefore, a lot of heat was produced in the polymerization reaction, which led to the high temperature of the system. This increased chain transfer and reduced the molecular weight, resulting in a decrease in hydrogel strength. When the content of montmorillonite was very high, a serious uneven distribution appeared in the system, which greatly reduced the effective crosslinking degree of the system and reduced the compressive strength [1,23].

### 3.3. Temperature Responsiveness of the Hydrogel

**Figure 4:**
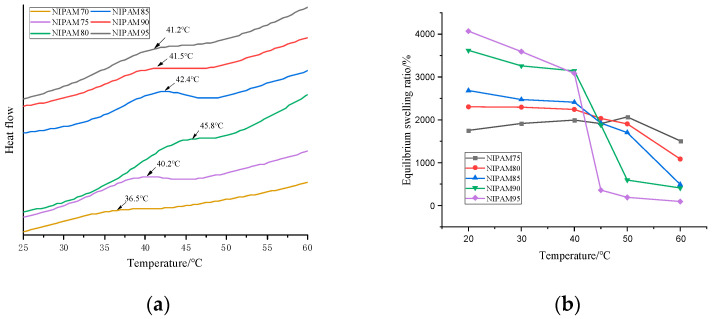
(**a**). Differential scanning calorimetry (DSC) curves of the GA–P(NIPAM-co-DMAPMA) hydrogels with different NIPAM contents. (**b**) Relationships of the equilibrium swelling ratio and the temperature for the GA–P(NIPAM-co-DMAPMA) hydrogels with different NIPAM contents.

The phase transition of the thermosensitive hydrogel occurred at a critical temperature. From the DSC curve of Figure 4a, it can be seen that the LCST of the hydrogel was affected by the NIPAM content. When the content of NIPAM in the monomer increased from 70% to 80%, the LCST of the hydrogel increased from 36.5 to 45.8 °C, which was higher than that of pure PNIPAM (32 °C) [32]. When the NIPAM concentration was higher than 80 wt%, the LCST decreased with the increase of concentration [33]. The critical phase transition mechanism of the PNIPAM hydrogel was due to the damage of hydrogen bonds and the enhancement of hydrophobic interaction [27]. The LCST of the series GA–P(NIPAM-co-DMAPMA) hydrogel was higher than that of the PNIPAM hydrogel, which may be due to the hydrophilic structure of GA with a large number of hydroxyl groups. The more the hydrophilic groups contained in the sample, the stronger the hydrogen bonding effect was. Therefore, it is necessary to destroy these hydrogen bonds at higher temperatures, so that the phase transformation can occur.

The glass transition temperature of the hydrogels increased from 118 to 142.4 °C with the increase of the NIPAM content in the monomer (Figure S3a), which was close to the glass transition temperature of the pure PNIPAM hydrogel (142 °C) [34]. GA had little effect on the glass transition temperature of the hydrogels, but there was an obvious weightlessness peak in the thermogravimetric curve of the hydrogels (Figure S3b). There were four peaks in the thermogravimetric curve of the hydrogel. The third stage was 272.81 to 387.62 °C, which was 7.402% of the weight loss due to the glycosidic bond broken in the sugar ring of GA. The fourth stage was from 387.62 to 437.06 °C, and the carbon–carbon bond of PNIPAM was broken. The polymer skeleton was decomposed, and the weight loss was 74.175%.

The GA–P(NIPAM-co-DMAPMA) hydrogel exhibited a temperature-sensitive feature. The equilibrium swelling ratios at different temperatures were important parameters for the performance of hydrogels. It can be seen from Figure 4b that when the content of NIPAM was 95 wt%, the material had the best thermal response and the highest equilibrium swelling ratio. In the temperature range of 20 to 65 °C, in addition to the significant change of transmittance (Figure 1), the equilibrium swelling ratio decreased with the increase of temperature, and the equilibrium swelling ratio decreased rapidly near the LCST (Figure 4b). When the temperature was lower than the LCST, the hydrophilic group and the water molecule form hydrogen bond, as well as the polymer chain, stretched, and the hydrogel absorbed water better. Thus, the equilibrium swelling ratio was greater. When the temperature was higher than the LCST, the hydrogen bonds between polymer chains were destroyed. The hydrophobic effect dominated the hydrogel, and the absorption energy of polymer chains was close to each other, resulting in the smaller volume of the hydrogel and the discharge of water and thus reducing the equilibrium swelling ratio. Figure 4b shows the equilibrium swelling ratios of the series GA–P(NIPAM-co-DMAPMA) hydrogel at 20–60 °C. The equilibrium swelling ratio of the hydrogel at 20 °C was relatively high, but with the increase of temperature, the equilibrium swelling ratio decreased and the equilibrium swelling rate decreased significantly after reaching the LCST. The possible reason for the deformation of the hydrogel is that when the ambient temperature changed, the interaction between the polymer chain and water molecules, the isopropyl group, and the amide group changed accordingly, breaking the original equilibrium state and causing the volume transformation of the hydrogel. When the temperature was below the LCST, the hydrogel absorbed water and expanded. When the hydrogel was above the LCST, the hydrophobicity of the isopropyl group on the GA–P(NIPAM-co-DMAPMA) chain was gradually enhanced and the hydrogen bonds between the amide group and the water molecule were also destroyed. The hydrophobic effect dominated, and the polymer chain began to aggregate, causing the gel network to shrink and dissolve.

### 3.4. Water Absorption Properties of theHydrogel

**Figure 5:**
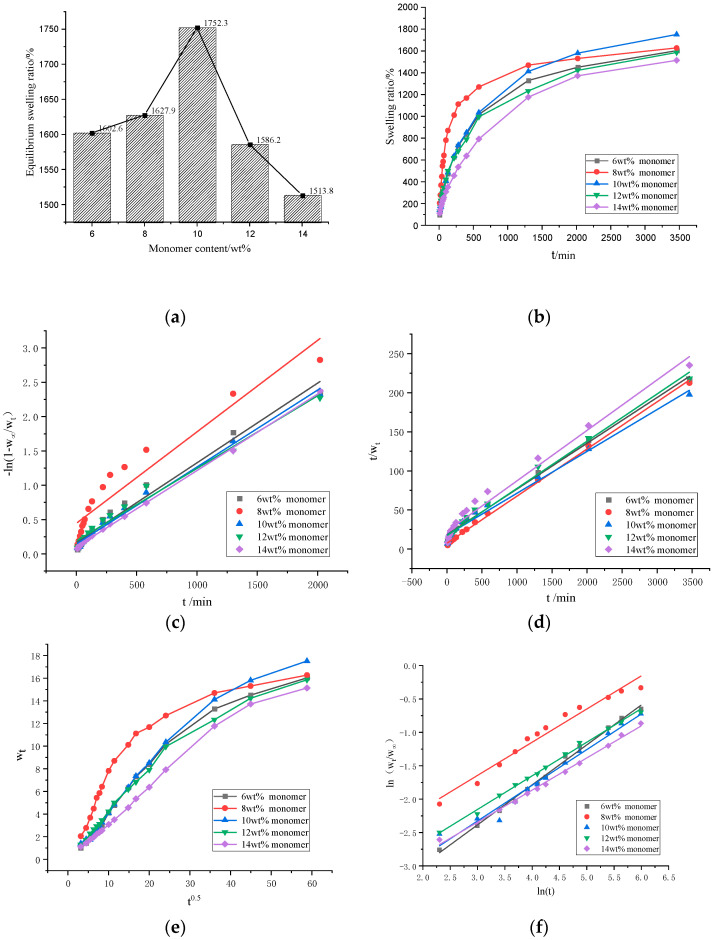
(**a**) The equilibrium swelling ratios of the GA–P(NIPAM-co-DMAPMA) hydrogels with different monomer contents. (**b**) Swelling kinetics curves of the hydrogels with different monomer contents. (**c**) Swelling kinetics of the hydrogels with different monomer contents fitted with the first-order kinetic equation. (**d**) Swelling kinetics of the hydrogels with different monomer contents fitted with the second-order kinetic equation. (**e**) Relationships between the swelling rate and t^0.5^ of hydrogel with different monomer concentration (**f**) (ln (W_t_/W_∞_)–lnt diagram) of the hydrogels with different monomer contents.

Figure 5c,d is the swelling tests results of the GA–P(NIPAM-co-DMAPMA) hydrogels with different monomer contents. The data were fitted by the first-order kinetic equation (Figure 5c) and fitted by the second-order kinetic equation (Figure 5d). The equations of the first-order dynamic model are shown as Equations (3)–(5):

(3) $$\frac{\mathrm{dW}}{\mathrm{dt}}=k_{w}\left(W_{\infty }-W_{t}\right),$$

(4) $$\ln\frac{W_{\infty }}{W_{\infty }-W_{t}}=k_{w}t,$$

(5) $$k_{w}t=-\ln\left(1-\frac{W_{t}}{W_{\infty }}\right),$$

where the value of k_w_ is the constant obtained by the ratio of the swelling rate to the swelling capacity and represents the water absorption rate in the first-order swelling; W_t_ is the swelling rate at time t; W_∞_ is the equilibrium swelling ratio; W′_∞_ is the theoretical equilibrium swelling ratio. The corresponding time t is plotted by −ln(1−W_t_/W_∞_). Under the circumstance that takes any value, if the corresponding swelling rate is proportional to its remaining swelling capacity, the swelling kinetics of hydrogels is a first-order kinetic model.

The corresponding time t (unit: min) was fitted by t/W_t_. If the correlation coefficient was greater than 0.95, the calculated W′_∞_ was close to the equilibrium swelling rate (W_∞_) calculated from the experimental data. This indicated that the pseudo-second-order kinetic model developed by Schott can effectively evaluate the swelling kinetics parameters of the hydrogels [35]. Schott’s second-order dynamic model is shown as Equations (6)–(8):

(6) $$\frac{\mathrm{dW}}{\mathrm{dt}}=k_{s}\left(W_{\infty }-W\right),$$

(7) $$\frac{t}{W}=A+\mathrm{Bt},$$

(8) $$A=\frac{1}{k_{s}w_{\infty }^{2}},$$

where B stands for 1/W_∞_; A is the reciprocal of the initial swelling ratio; K_s_ is the swelling ratio constant. The R^2^ value of the linear fitting is proportional to the degree of fitting. Therefore, by comparing the R^2^ value of the linear equation of the first-order kinetic model of the same crosslinking agent with that of the second-order kinetic model, we can judge which model is more suitable for the swelling kinetics of the sample hydrogel.

Figure 5a,b shows the isothermal swelling kinetics curves of the GA–P(NIPAM-co-DMAPMA) hydrogels. When the monomer content was 10 wt%, the hydrogel had the largest equilibrium swelling ratio of 1752.3%. The series of hydrogels absorbed water rapidly in the first 10 h and slowly in the later stage. The increase of the NIPAM content enhanced the sensitivity of the hydrogel to temperature (Figure S4a–j). The hydrogel had a higher equilibrium swelling ratio at 20 °C and a lower equilibrium water absorption at 60 °C. Especially, the higher the NIPAM content was, the stronger the temperature sensitivity of equilibrium swelling ratio was (Table S1).For example, when the NIPAM content was 95%, the equilibrium swelling ratios of the hydrogel were 4070% at 20 °C and 93% at 60 °C. When the monomer content was higher than 10 wt%, the swelling kinetics of the sample followed the first-order kinetics (Figure 5c and Table S2).When the monomer content was lower than 10 wt%, the second-order equation had a higher linear fitting degree. The swelling process of the hydrogel accorded with the second-order kinetic model (Figure 5d and Table S3). When the content of the initiator and the crosslinker was 2wt%, the maximum equilibrium swelling ratio was 1795% (Figure S5a,b), the linear fitting degree of the second-order equation was high (Figure S5c,d, Tables S5 and S6). When the content of initiator was 5 wt%, the maximum equilibrium swelling ratio was 1829% (Figure S6a,b), the second-order equation had a high degree of linear fitting, and the swelling process of the hydrogel accorded with the second-order kinetic model (Figure S6c,d, Tables S8 and S9).

It can be seen from Figure 5e that the molecular chain relaxation of hydrogels dominated its swelling rate during this swelling process, regardless of the monomer content. When the monomer content was 8wt%, the bending degree was largest, which indicated that the diffusion rate of water molecules was far greater than the relaxation rate of polymer segments.

In order to quantitatively judge the diffusion type and analyze the diffusion behavior accurately, we took the swelling data with the swelling rate and the equilibrium swelling rate no higher than 60% for further analysis and linear fitting. We used the Ritger–peppas model to describe the swelling mechanism of hydrogels at the initial stage. The model can be expressed by Equations (9) and (10):

(9) $$\frac{W_{t}}{W_{\infty }}={\mathrm{kt}}^{n},$$

(10) $$\ln\left(\frac{W_{t}}{W_{\infty }}\right)=\mathrm{nlnt}+\mathrm{lnk}$$

where the swelling rate at t (min) is expressed by W_t_, W_∞_ is the equilibrium swelling rate, K is a hydrogel characteristic constant related to the network structure, and n is the diffusion index that determines the diffusion mechanism of water molecules.

Using lnt as the x coordinate and ln(W_t_/W_∞_) as the y coordinate, the scatter plots of series of the hydrogels with different monomer contents were made, and a straight fitting line was established. The constants *n* and K can be calculated from the slope and intercept of the ln(W_t_/W_∞_)-to-lnt fitting line graph. The fitted linear equation of the series of the hydrogels had a good linear correlation coefficient (R^2^ > 0.95), and from Figure 5f, it can be seen that the degree of fitting was relatively high. When the monomer contentswere 8, 12, and 14 wt%, the values of *n* were between 0.45 and 0.5 (Table S4), which indicated that the diffusion behavior of the hydrogel belongs to Fickian swelling. When the monomer contents were 6% and 10%, *n* was larger than 0.5 (Table S4), indicating that the diffusion behavior of the sample belongs to non-Fickian swelling, and the diffusion rate of water molecule was equivalent to the rate of chain relaxation. When the monomer content was 6wt%, the *n* value was 0.60, that is, the fastest diffusion rate of hydrogel was generated, probably because when the monomer content was low, the degree of crosslinking was low, and the structure of hydrogel was loose. When the content of initiator and crosslinker was 2 wt%, the *n* value of the hydrogel was 0.67 (0.5 < *n* < 1) (Figure S3f, Table S7). When the content of the initiator was 5 wt%, the *n* value of the hydrogel was 0.53 (0.5 < *n* < 1) (Figure S4e–f, Table S10), which indicated that the diffusion behavior of the hydrogel belonged to non-Fickian swelling (Figure S4f, Table S10).

### 3.5. Drug-Controlled Release Behavior of theHydrogels

**Figure 6:**
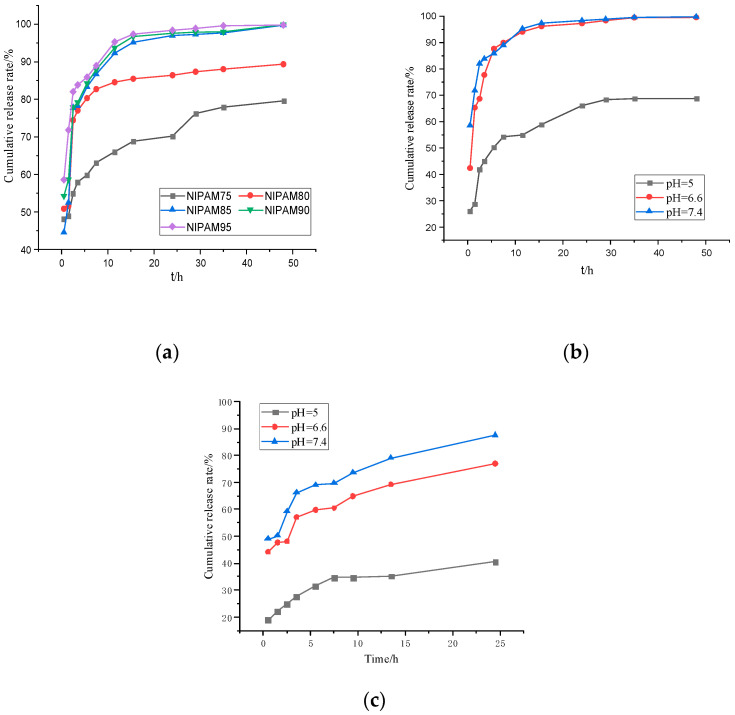
(**a**) The cumulative release rate of the encapsulated bovine serum albumin GA–P(NIPAM-co-DMAPMA) hydrogel. (**b**) The cumulative release rate of bovine serum albumin hydrogel in a buffer solution of pH = 7.4 at 37 °C. (**c**) The cumulative release rate of bovine serum albumin hydrogel varied with time at 25°C.

The temperature-sensitive and pH-sensitive properties of the GA–P(NIPAM-co-DMAPMA) hydrogel made it an ideal drug carrier. The drug release behavior of the hydrogel was investigated by using bovine serum albumin as a model drug (Figure 6). Figure 6a shows the change of the BSA cumulative release rate of the GA–P(NIPAM-co-DMAPMA) hydrogel containing different NIPAM contents in a buffer solution at 37 °C and pH 7.4. It can be seen from Figure 6a that with the increase of NIPAM monomer content, the early release rate of bovine serum albumin was faster and the cumulative release rate was larger. NIPAM had a great effect on the shrinkage/swelling behavior of hydrogels. The difference of the equilibrium swelling ratio of the GA–P(NIPAM-co-DMAPMA) hydrogel samples between 20 and 60 °C can reach 3977%, when the content of NIPAM was 95 wt% (Table S1), which allowed the hydrogel to release a large amount of adsorption liquid when the temperature changed. It can be seen that the hydrogel with high NIPAM content had high volume phase transformation and shrinkage and can adsorb or squeeze more water (Figure 4b). The GA–P(NIPAM-co-DMAPMA) hydrogel was used as a drug carrier to load drugs, and bovine serum albumin entered the hydrogel under osmotic pressure. Secondly, the drug extruded the macromolecular bovine serum albumin through volume transformation and contraction. The hydrogel with high NIPAM content had high shrinkage. Therefore, the content of NIPAM had great influence on the drug release rate and cumulative release amount. The release rate of the hydrogel in pH7.4 was fast, and the total amount of release was the highest (Figure 6b,c).This is because the GA–P-(NIPAM-co-DMAPMA) hydrogel contained amino groups and the dissociation of the basic groups in the polymer chain increased with the decrease of pH. The corresponding hydrogel and water molecules weakened the formation of hydrogen bonds, resulting in the decrease of the swelling ability of hydrogels. Therefore, the water absorption rates of the hydrogels were different at different pH values, leading to differences in hydrogel characteristics, which indicated the pH sensitivity of the hydrogel.

## 4. Conclusions

In this study, the multifunctional GA–P(NIPAM-co-DMAPMA) hydrogel was successfully prepared by free radical aqueous copolymerization. The stress and elastic modulus of the hydrogel containing GA were higher than those of the hydrogel without GA. Controlling the content of NIPAM in the hydrogels can regulate the LCST value in a certain range. When the temperature increased, the molecular network of the hydrogel decreased, the transmittance decreased, the temperature decreased, the hydrogel expanded, and the transmittance increased. The difference of the equilibrium swelling ratio of the GA–P(NIPAM-co-DMAPMA) hydrogel samples between 20 and 60 °C can reach 3977%. With the increase of NIPAM monomer content, the early release rate of bovine serum albumin was faster, and the cumulative release rate was larger. The release rate of the hydrogel in pH7.4 was fast, and the total amount of release was the highest. The hydrogel has the characteristics of temperature and pH response and has good controlled release ability to bovine serum albumin.The GA–P(NIPAM-co-DMAPMA) hydrogel is a potential carrier material for colon targeting drug delivery.

## Figures and Tables

**Table 1:** GA–P(NIPAM-co-DMAPMA) hydrogel experimental scheme.

Sample	GA(g)	NIPAM (g)	DMAPMA (g)	Montmorillonite (g)	H_2_O (mL)
NIPAM 70	0.05	0.791	0.51	0.2	10
NIPAM 75	0.05	0.848	0.425	0.2	10
NIPAM 80	0.05	0.906	0.34	0.2	10
NIPAM 85	0.05	0.96	0.255	0.2	10
NIPAM 90	0.05	1.017	0.17	0.2	10
NIPAM 95	0.05	1.075	0.086	0.2	10
NIPAM 100	0.05	1.2	0	0.2	10

## Data Availability

The data presented in this study are available on request from the corresponding author.

## Supplementary Materials

The following are available online at https://www.mdpi.com/2073-4360/13/1/119/s1, Figure S1: Schematic diagram of GG graft copolymerization., Figure S2: Mechanical strength of GA-P (NIPAM co DMAPMA) hydrogel (a) initiator content; (b) monomer content, (c) montmorillonite content., Figure S3: DSC and TGA curves of GA-P (NIPAM-DMAPMA) hydrogels ((a) DSC curve; (b) TGA curve), Figure S4: The swelling kinetic curve and Equilibrium swelling ratio of GA-P (NIPAM-DMAPMA) hydrogel with different NIPAM monomer ratios ((a) 60 °C; (b) 60 °C; (c) 50 °C; (d) 50 °C; (e) 40 °C; (f) 40 °C; (g) 30 °C; (h) 30 °C; (i) 20 °C; (j) 20 °C), Figure S5: GA-P (NIPAM-DMAPMA) hydrogels with different crosslinking agent content ((a) Swelling kinetic curve; (b) Equilibrium swelling ratio; (c) Fitting curve of kinetic equation of first-order kinetics; (d) second-order kinetics; (e) Relation between swelling rate and t0.5; (f) ln(Wt/W∞)~ lnt relationship), Figure S6: GA-P (NIPAM-DMAPMA) hydrogels with different initiator (APS) content ((a) Swelling kinetic curve; (b) Equilibrium swelling ratio; (c) Fitting curve of kinetic equation of first-order kinetics; (d) second-order kinetics; (e) Relation between swelling rate and t0.5; (f) ln(Wt/W∞)~ lnt relationship), Table S1: The equilibrium swelling ratio (ESR) of GG-PNIPAM composite hydrogel at different temperatures, Table S2: The first-order kinetic model parameters of the GA–P(NIPAM-co-DMAPMA) hydrogels with different monomer contents, Table S3: The second-order kinetic model parameters of the GA–P(NIPAM-co-DMAPMA) hydrogels with different monomer contents, Table S4: Swelling kinetic parameters and fitting equation of the GA–P(NIPAM-co-DMAPMA) hydrogels with different monomer contents, Table S5: Parameters of the first-order kinetics model of the GA–P(NIPAM-co-DMAPMA) hydrogels with different crosslinking agent contents, Table S6: Second-order kinetic model parameters of the GA–P(NIPAM-co-DMAPMA) hydrogels with different crosslinking agent contents, Table S7: Swelling kinetic parameters and fitting equation of the GA–P(NIPAM-co-DMAPMA) hydrogels with different crosslinking agent (MBA)contents, Table S8: Parameters of first-order kinetics model of the GA–P(NIPAM-DMAPMA) hydrogels with different initiator (APS) contents, Table S9: Parameters of the second-order kinetics model of the GA–P(NIPAM-co-DMAPMA) hydrogels with different initiator (APS) contents, Table S10: Swelling kinetic parameters and fitting equation of the GA–P(NIPAM-DMAPMA) hydrogels with different initiator (APS) contents.
