# Supplementary material for: Preparation and Drug Release Properties of a Thermo Sensitive GA Hydrogel
Source: Polymers (Basel). 2020 Dec 30;13(1):119. doi: 10.3390/polym13010119 (PMC7796085; doi:10.3390/polym13010119)
Supplement: Supplementary file 1 [file polymers-13-00119-s001.pdf]

polymers

Supplementary Materials

Preparation and Drug Release Properties of a Thermo Sensitive GA Hydrogel

Jiufang Duan *, Yirong Huang, Shiyu Zong and Jianxin Jiang

MOE Engineering Research Center of Forestry Biomass Materials and Bioenergy, Beijing Forestry University, Beijing 100083, China; huangyirong@163.com (Y.H.); zongshiyu@126.com (S.Z.); jiangjx@bjfu.edu.cn (J.J.)
* Correspondence: duanjiuf@bjfu.edu.cn

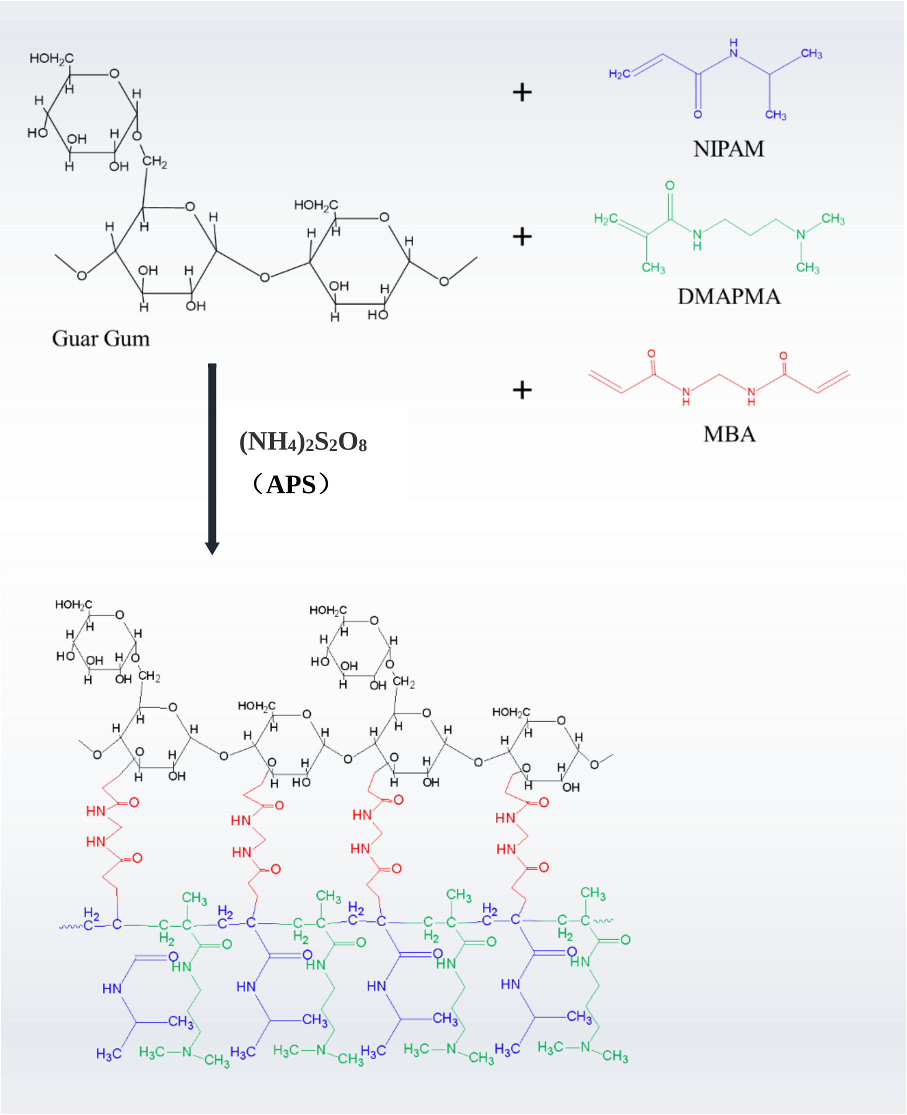

Figure S1. Schematic diagram of GG graft copolymerization.

Citation: Duan, J.; Huang, Y.; Zong, S.; Jiang, J. Preparation and drug release properties of a thermo sensitive GA hydrogel. *Polymers* **2021**, *13*, 119. https://doi.org/10.3390/polym13010119

Received: 10 December 2020
Accepted: 25 December 2020
Published: 30 December 2020

Publisher's Note: MDPI stays neutral with regard to jurisdictional claims in published maps and institutional affiliations.

Copyright: © 2020 by the authors. Submitted for possible open access publication under the terms and conditions of the Creative Commons Attribution (CC BY) license (http://creativecommons.org/licenses/by/4.0/).

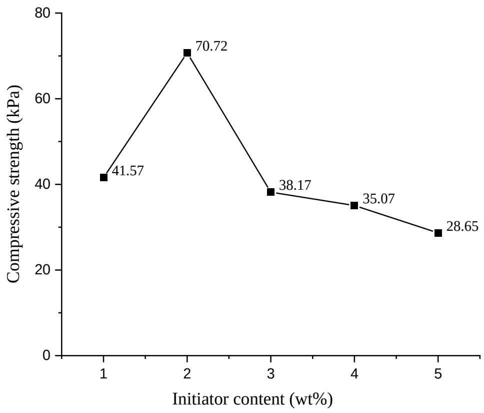

(**a**)

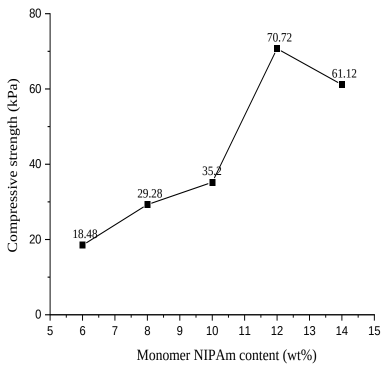

(**b**)

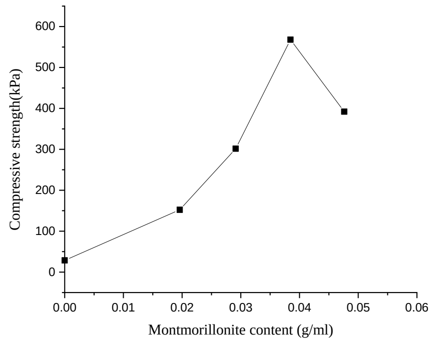

(**c**)

Figure S2. Mechanical strength of GA-P (NIPAM co DMAPMA) hydrogel (**a**) initiator content; (**b**) monomer content, (**c**) montmorillonite content.

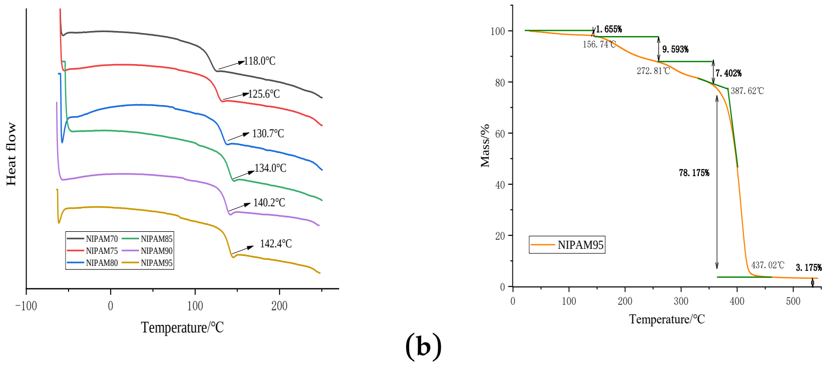

(**a**) (**b**)

Figure S3. DSC and TGA curves of GA-P (NIPAM-DMAPMA) hydrogels ((**a**) DSC curve; (**b**) TGA curve).

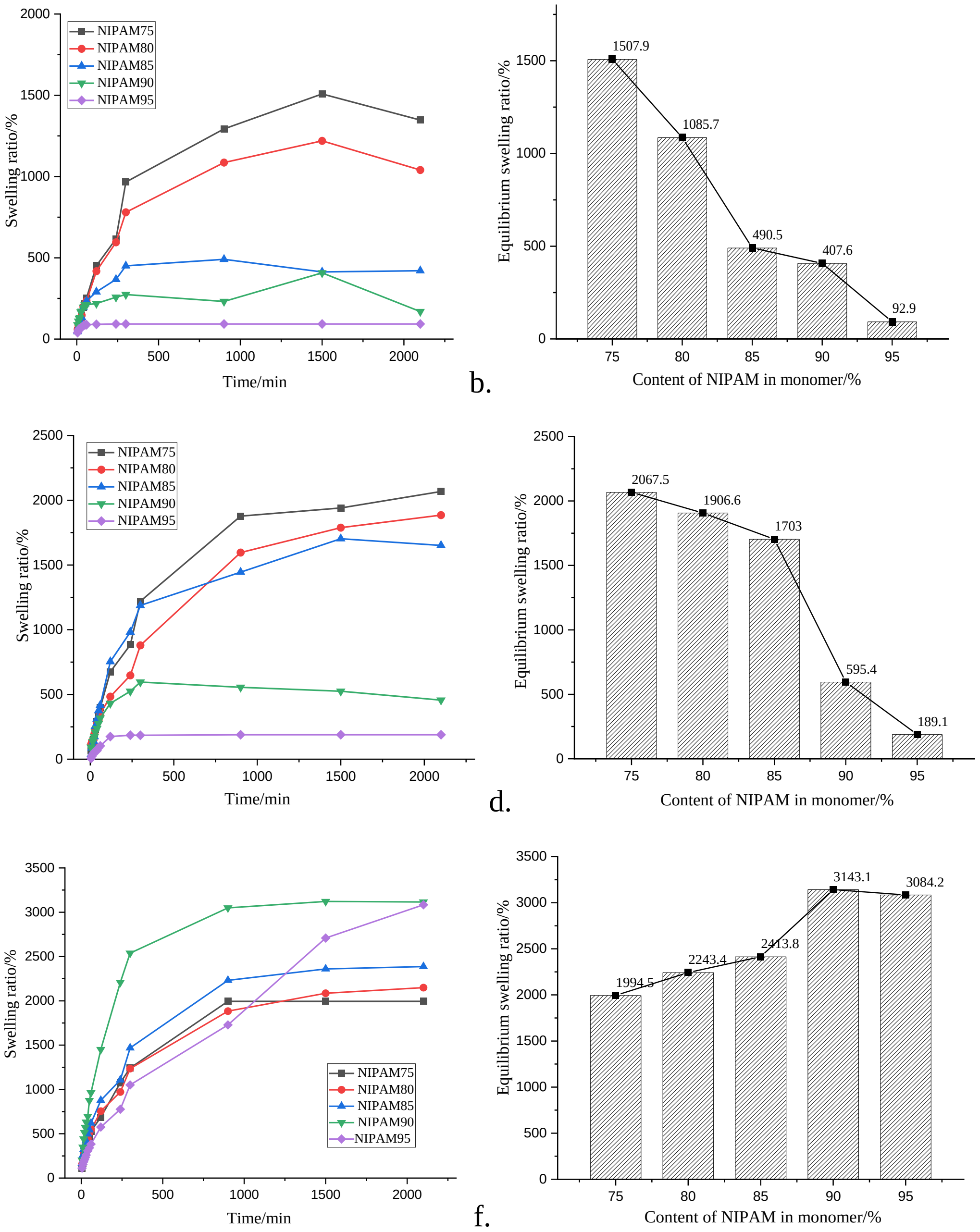

a. b.

c. d.

e. f.

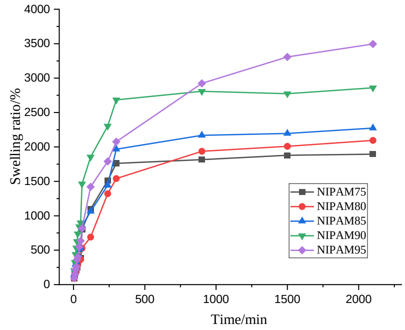

g.

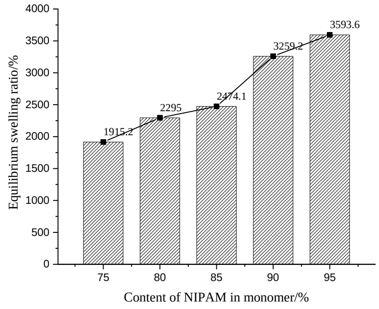

h.

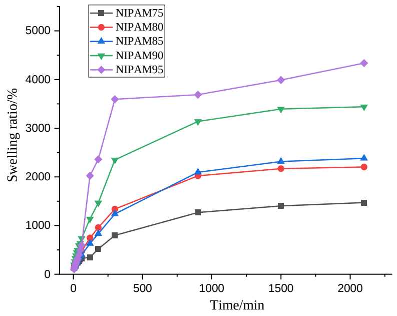

i.

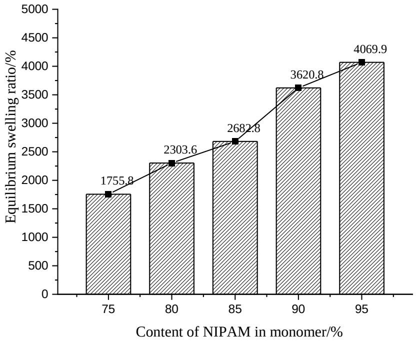

j.

Figure S4. The swelling kinetic curve and Equilibrium swelling ratio of GA-P (NIPAM-DMAPMA) hydrogel with different NIPAM monomer ratios ((**a**) 60 °C; (**b**) 60 °C; (**c**) 50 °C; (**d**) 50 °C; (**e**) 40 °C; (**f**) 40 °C; (**g**) 30 °C; (**h**) 30 °C; (**i**) 20 °C; (**j**) 20 °C).

Table S1. The equilibrium swelling ratio (ESR) of GG-PNIPAM composite hydrogel at different temperatures.

**Sample**	**$ESR_{20℃}$ / %**	**$ESR_{30℃}$ / %**	**$ESR_{40℃}$ / %**	**$ESR_{45℃}$ / %**	**$ESR_{50℃}$ / %**	**$ESR_{60℃}$ / %**	**($ESR_{60℃}$ -$ESR_{20℃}$) / %**
NIPAM 75	1756	1915	1995	1911	1808	1508	248
NIPAM 80	2304	2295	2243	2033	1907	1086	1218
NIPAM 85	2683	2474	2414	1924	1703	491	2192
NIPAM 90	3621	3259	3143	1883	595	408	3213
NIPAM 95	4070	3594	3084	359	148	93	3977

Table S2. The first-order kinetic model parameters of GA-P (NIPAM-DMAPMA) hydrogel with different monomer contents.

**Monomer content /wt%**	**Linear fitting equation**	**R^2**	**k**
6	y=0.0012x + 0.1641	0.979	0.0012
8	y=0.0013x + 0.4439	0.913	0.0013
10	y=0.0011x + 0.1432	0.991	0.0011
12	y=0.0011x + 0.1863	0.982	0.0011
14	y=0.0011x + 0.0992	0.999	0.0011

Table S3. The second-order kinetic model parameters of GA-P (NIPAM-DMAPMA) hydrogel with different monomer contents.

Monomer content /wt%	Linear fitting equation	R^2	k_s	W'_∞	W_∞
6	y=0.0585x + 18.289	0.994	0.00019	17.09402	16.02637
8	y=0.0603x + 7.3732	0.999	0.00049	16.58375	16.27924
10	y=0.0534x + 18.068	0.989	0.00016	18.72659	17.52275
12	y=0.0604x + 17.158	0.989	0.00021	16.55629	15.86245
14	y=0.0646x + 22.659	0.979	0.00018	15.47988	15.13802

Table S4. Swelling kinetic parameters and fitting equation of GA-P (NIPAM-DMAPMA) hydrogels with different monomer contents.

monomer contents /%	Linear fitting equation	R^2	n	k
6	y=0. 5985x−4. 1799	0. 996	0. 60	−4. 18
8	y=0. 4968x−3. 1345	0. 970	0. 50	−3. 13
10	y=0. 5340x−3. 9282	0. 977	0. 53	−3. 93
12	y=0. 5032x−3. 6632	0. 996	0. 50	−3. 66
14	y=0. 4808x−3. 7871	0. 996	0. 48	−3. 79

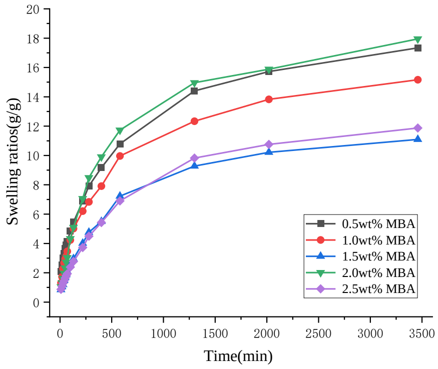

a.

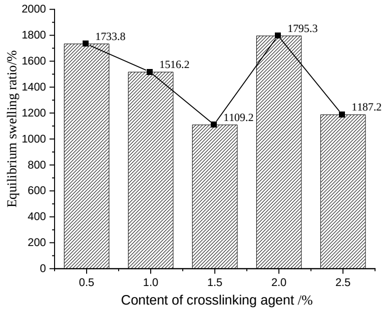

b.

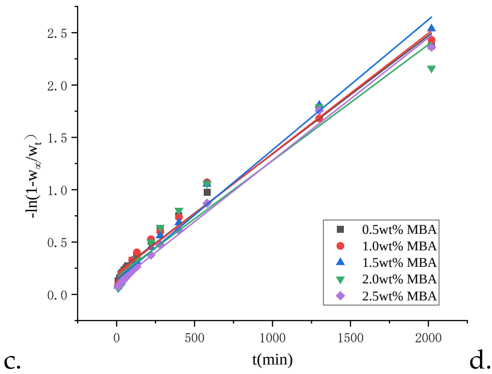

c.

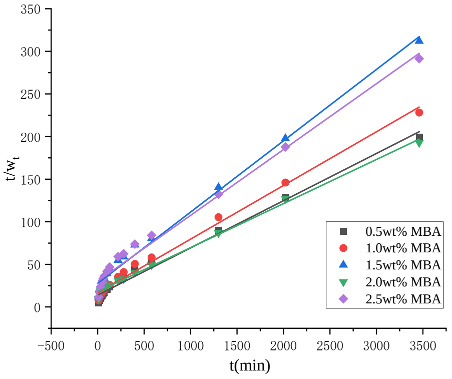

d.

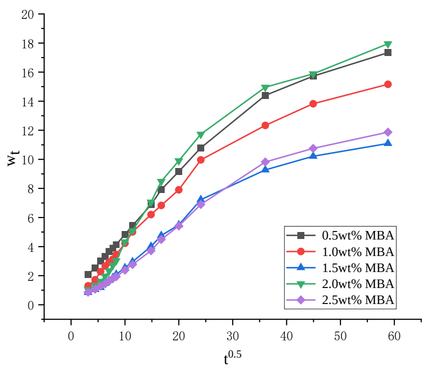

e.

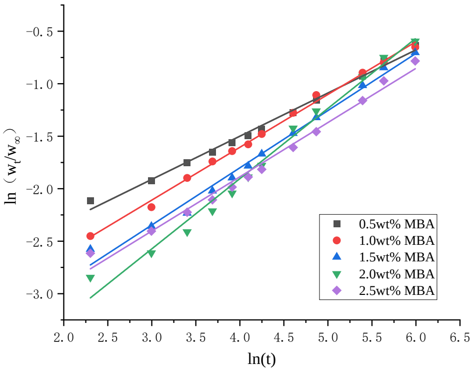

f.

Figure S5. GA-P (NIPAM-DMAPMA) hydrogels with different crosslinking agent content ((**a**) Swelling kinetic curve; (**b**) Equilibrium swelling ratio; (**c**) Fitting curve of kinetic equation of first-order kinetics; (**d**) second-order kinetics; (**e**) Relation between swelling rate and $t^{0.5}$; (**f**) $\ln(W_t/W_\infty)$~lnt relationship).

Table S5. parameters of first-order kinetics model of GA-P (NIPAM-DMAPMA) hydrogels with different crosslinking agent content.

Crosslinking agent content /wt%	Linear fitting equation	R^2	K_w
0.5	y=0.0011x + 0.209	0.988	0.0011
1	y=0.0011x + 0.1959	0.983	0.0011
1.5	y=0.0012x + 0.1364	0.989	0.0012
2	y=0.0011x + 0.1668	0.951	0.0011
2.5	y=0.0012x + 0.1078	0.993	0.0012

Table S6. second-order kinetic model parameters of GA-P (NIPAM-DMAPMA) hydrogels with different crosslinking agent content.

Crosslinking agent content /wt%	Linear fitting equation	R^2	k_s	W'_∞	W_∞
0.5	y=0.0554x + 13.896	0.989	0.00022	18.05054	17.33826
1	y=0.0630x + 16.618	0.992	0.00024	15.87302	15.16245
1.5	y=0.0836x + 27.876	0.992	0.00025	11.96172	11.09213
2	y=0.0517x + 17.887	0.996	0.00015	19.34236	17.95264
2.5	y=0.0773x + 30.241	0.985	0.00020	12.93661	11.87250

Table S7. Swelling kinetic parameters and fitting equation of GA-P (NIPAM-DMAPMA) hydrogels with different crosslinking agent (MBA)content.

Crosslinking agent content /wt%	Linear fitting equation	R^2	n	k
0.5	y=0.4113x - 3.1451	0.993	0.41	-3.15
1	y=0.5032x - 3.6181	0.996	0.50	-3.62
1.5	y=0.5451x - 3.9813	0.989	0.55	-3.99
2	y=0.6691x - 4.5806	0.986	0.67	-4.58
2.5	y=0.5167x - 3.9534	0.987	0.52	-3.95

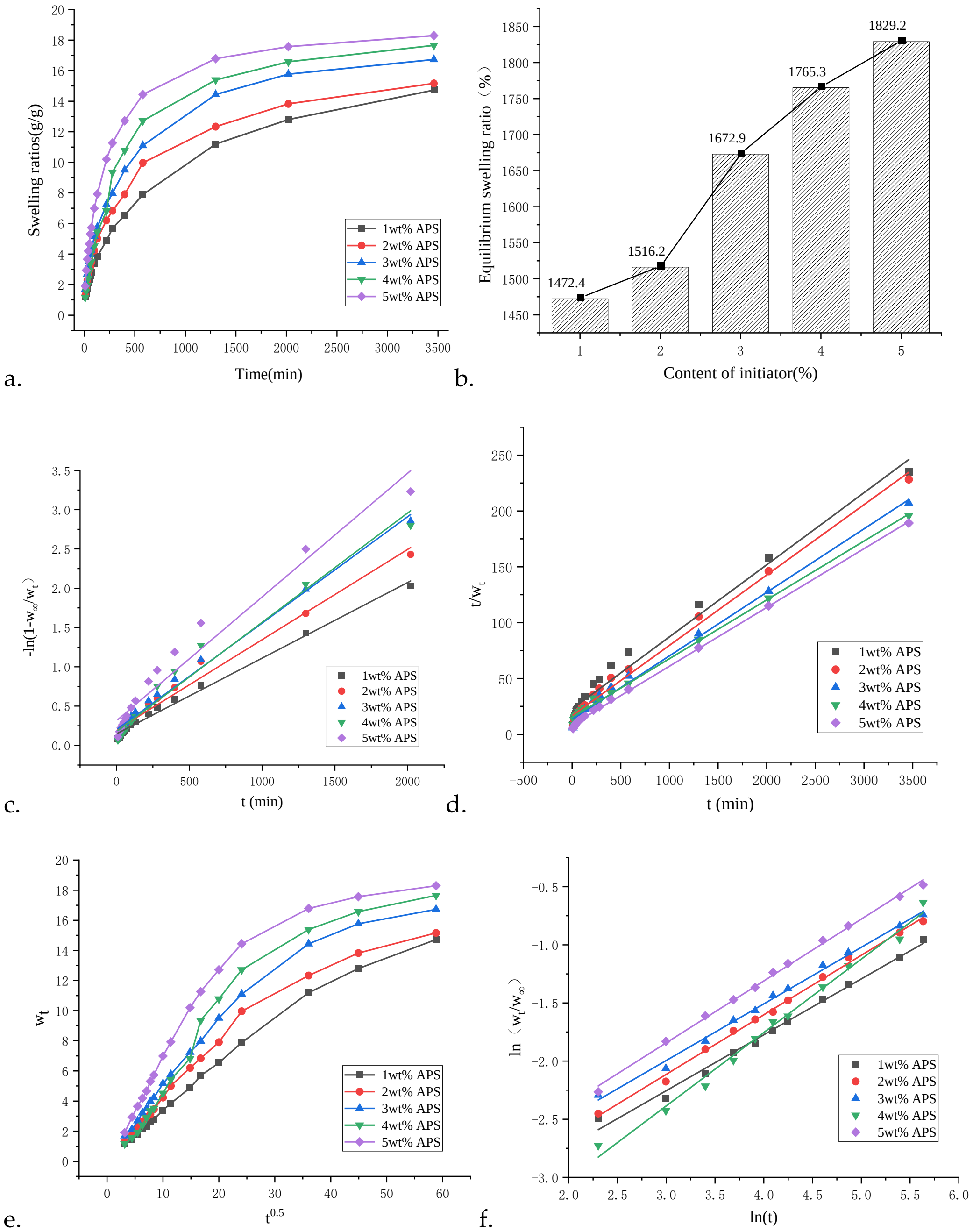

Figure S6. GA-P (NIPAM-DMAPMA) hydrogels with different initiator (APS) content((**a**) Swelling kinetic curve; (**b**) Equilibrium swelling ratio; (**c**) Fitting curve of kinetic equation of first-order kinetics; (**d**) second-order kinetics; (**e**) Relation between swelling rate and $t^{0.5}$; (**f**) $\ln(W_t/W_\infty)$~ lnt relationship).

Table S8. Parameters of first-order kinetics model of GA-P (NIPAM-DMAPMA) hydrogels with different initiator (APS) content.

Initiator content /wt%	Linear fitting equation	R^2	k
1	y=0.0010x + 0.1469	0.993	0.001
2	y=0.0011x + 0.1959	0.983	0.0011
3	y=0.0014x + 0.2007	0.992	0.0014
4	y=0.0014x + 0.1744	0.972	0.0014
5	y=0.0016x + 0.3141	0.962	0.0016

Table S9. Parameters of second-order kinetics model of GA-P (NIPAM-DMAPMA) hydrogels with different initiator (APS) content.

Initiator content /wt%	Linear fitting equation	R^2	K_s	W'_∞	W_∞
1	y=0.0646x + 22.659	0.979	0.00018	15.47988	14.72429
2	y=0.0630x + 16.618	0.992	0.00024	15.87302	15.16245
3	y=0.0569x + 13.289	0.994	0.00024	17.57469	16.72857
4	y=0.0527x + 15.018	0.997	0.00019	18.97533	17.65313
5	y=0.0526x + 8.3055	0.999	0.00033	19.01141	18.29176

Table S10. Swelling kinetic parameters and fitting equation of GA-P (NIPAM-DMAPMA) hydrogels with different initiator (APS) content.

Initiator content /wt%	Linear fitting equation	R^2	n	k
1	y=0.4808x - 3.6971	0.992	0.48	-3.70
2	y=0.5121x - 3.6505	0.997	0.51	-3.65
3	y=0.4868x - 3.4553	0.996	0.49	-3.46
4	y=0.6311x - 4.2787	0.991	0.63	-4.29
5	y=0.5320x - 3.4389	0.998	0.53	-3.44
